# Current perspectives on the mechanisms of auditory hallucinations: introduction to the special research topic

**DOI:** 10.3389/fnhum.2013.00792

**Published:** 2013-11-18

**Authors:** Johanna C. Badcock, Frank Larøi, Kelly Diederen, Paul Allen

**Affiliations:** ^1^School of Psychology, University of Western AustraliaCrawley, WA, Australia; ^2^Department of Psychology, Cognition and Behaviour, University of LiègeLiège, Belgium; ^3^Department of Physiology, Development and Neuroscience, University of CambridgeCambridge, UK; ^4^Institute of Psychiatry, King's CollegeLondon, UK

**Keywords:** auditory hallucinations, cognition, emotion, hearing voices, non-clinical hallucinations, psychosis, schizophrenia

According to the Oxford English Dictionary (http://www.oed.com) the term “perspective” is derived from the verb *perspicere* (from *per*- “through” + *specere* “to look”), meaning “looked at closely.” In keeping with this origin, the contributors to this Frontiers Research Topic have indeed *looked closely* at the experience of auditory hallucinations (AH), providing new insights into the precise nature of hallucinations in clinical and non-clinical groups; the underlying cognitive, emotional and neural processes; and how this evidence might inform the next generation of personalized treatments Connor and Birchwood (2013).

The phenomenology of AH and the mechanisms that underpin them lie at the heart of our research, yet there has been a growing concern that the characteristics of AH prioritized by researchers don't match those considered important to hallucinators themselves. To some degree this disparity reflects the (sometimes) limited scope of popular research assessment tools. However, disregarding the phenomenological diversity of AH may guarantee that we will fail to understand the biopsychosocial processes involved. Consequently, the resurgence of interest in phenomenological research reflected in current contributions is both timely and welcome. i, Broome and Fernyhough McCarthy-Jones et al. (2013) call for more nuanced descriptions of AH that reflect more accurately what the experience is like, and proffer a philosophical phenomenological perspective, which encourages a systematic exploration of (normal and AH) experiences from the first person perspective. Both Badcock and Chhabra (2013) and deLeede-Smith and Barkus (2013) tackle the diverse presentations of AH in clinical and non-clinical groups. deLeede-Smith and Barkus adopt a developmental perspective, charting how the features of AH emerge and persist across the lifespan, and suggest that mechanisms maintaining AH differ across these populations, whilst Badcock and Chhabra (2013) provide an extensive and reflective review of the literature on the perception of voice identity, which points to subtle biases across different levels of voice identity. Rounding out this set of articles, Stephane (2013) emphasizes the heterogeneity of AH from one voice hearer to the next, and persuasively argues that whilst hallucinatory experiences are unique to each individual they are not random collections of features. Instead, Stephane suggests, they arise from a limited number of dimensions of phenomenological AH space—each linked to a separate neural basis.

Continuing the neural processing theme, Kompus et al. (2013) show that while non-clinical voice hearers present with a reduced response to speech sounds in the primary auditory cortex, attentional modulation of this area is intact. In strong contrast, no attentional modulation of this area could be observed in schizophrenia patients with AH. Further similarities as well as differences in mechanisms underlying AH are shown by i Dahoun et al. (2013) who compared neural correlates of mentally simulated actions (e.g., “open a window”) between hallucination-prone adolescents and a group with a genetic risk for schizophrenia (22q11.2 deletion syndrome). While both groups exhibited decreased activation in regions related to self-other distinction when imagining a close friend performing an action, individuals with a genetic risk for schizophrenia displayed additional decreased activations in areas associated with visual imagery, episodic memory and social cognition when “simply” seeing a cue that said either “you” or “best friend” earlier on in the task.

Another set of articles highlight the role of emotion in AH. Kanemoto et al. (2013) show that university students with high hallucination proneness tended to make more external misattributions of inner thoughts than those with lower hallucinations proneness. Importantly, they also show that emotional valence affected the ability to recall whether a word had been previously heard, or had been imagined, only in the latter group of subjects. Similarly, with the help of an implicit emotional prosody task, Alba-Ferrara et al. (2013) examine how attention is involuntarily captured in patients with schizophrenia and healthy controls, and conclude that patients with AH may be less lateralized in their processing of emotion conveyed in voice. Finally, based on studies that have demonstrated that patients with schizophrenia have difficulty perceiving and discriminating emotions based on affective prosody compared to healthy controls, Tucker et al. (2013) wished to examine whether this may also represent an endophenotype of schizophrenia, i.e., by examining this capacity in non-affected, first-degree relatives. They found that unaffected relatives of AH schizophrenia patients, compared to the matched healthy controls, exhibited some basic impairments in auditory processing, suggesting that auditory processing deficits may be a core feature of AH in schizophrenia, but that the two groups did not differ significantly on a major variable, i.e., number of errors in pitch discrimination. Clearly, more research is needed in order to examine whether these difficulties represent a potential endophenotype for AH.

Finally, from a neurofunctional perspective, two papers report new findings in patients with schizophrenia and auditory verbal hallucinations (AVH). Homan et al. (2013) used Arterial Spin labeling (ASL)—a Magnetic Resonance technique for measuring cerebral blood flow (CBF)—to show that increased perfusion in the left superior temporal gyrus (STG; a cerebral area known to support language and auditory function) in patients with schizophrenia and AVH, relative to controls and global CBF in patients, was not reduced by treatment with transcranial magnetic stimulation even though AVH symptom scores decreased over the treatment period. These findings are consistent with what has previously been termed a trait marker of AVH in schizophrenia. The second paper by Koeda et al. (2013) used a novel functional MRI favorability judgment task to show that hypo-activation in the left STG may be associated with brain dysfunction in accessing vocal attractiveness in schizophrenia, although right fronto- parietal regions could offset STG dysfunction associated with social communication.

In sum, the variety of perspectives featured in this Research Topic illustrates the vitality of current research on AH across a range of diagnostic groups, and the significant advances that have been made in understanding the mechanisms the underlie them.

## References

[B1] Alba-Ferrara L. de Erausquin G. A. Hirnstein M. Weis S. Hausmann M. (2013). Emotional prosody modulates attention in schizophrenia patients with hallucinations. Front. Hum. Neurosci. 7:59 10.3389/fnhum.2013.00059 23459397 PMC3586698

[B2] Badcock J. C. Chhabra S. (2013). Voices to reckon with: perceptions of voice identity in clinical and non-clinical voice hearers. Front. Hum. Neurosci. 7:114 10.3389/fnhum.2013.00114 23565088 PMC3615181

[B3] Connor C. Birchwood M. (2013). Through the looking glass: self-reassuring meta-cognitive capacity and its relationship with the thematic content of voices. Front. Hum. Neurosci. 7:213 10.3389/fnhum.2013.00213 23734118 PMC3659289

[B4] Dahoun T. Eliez S. Chen F. Badoud D. Schneider M. Larøi F. (2013). Action simulation in hallucination-prone adolescents. Front. Hum. Neurosci. 7:329 10.3389/fnhum.2013.00329 23847502 PMC3701149

[B5] de Leede-Smith S. Barkus E. (2013). A comprehensive review of auditory verbal hallucinations: lifetime prevalence, correlates and mechanisms in healthy and clinical individuals. Front. Hum. Neurosci. 7:367 10.3389/fnhum.2013.00367 23882203 PMC3712258

[B6] Homan P. Kindler J. Hauf M. Walther S. Hubl D. Dierks T. (2013). Repeated measurements of cerebral blood flow in the left superior temporal gyrus reveal tonic hyperactivity in patients with auditory verbal hallucinations: a possible trait marker. Front. Hum. Neurosci. 7:304 10.3389/fnhum.2013.00304 23805093 PMC3691504

[B7] Kanemoto M. Asai T. Sugimori E. Tanno Y. (2013). External misattribution of internal thoughts and proneness to auditory hallucinations: the effect of emotional valence in the Deese–Roediger–McDermott paradigm. Front. Hum. Neurosci. 7:351 10.3389/fnhum.2013.00351 23847517 PMC3705193

[B8] Koeda M. Takahashi H. Matsuura M. Asai K. Okubo Y. (2013). Cerebral responses to vocal attractiveness and auditory hallucinations in schizophrenia: a functional MRI study. Front. Hum. Neurosci. 7:221 10.3389/fnhum.2013.00221 23745111 PMC3662879

[B9] Kompus K. Falkenberg L. E. Bless J. J. Johnsen E. Kroken R. A. Kråkvik B. (2013). The role of the primary auditory cortex in the neural mechanism of auditory verbal hallucinations. Front. Hum. Neurosci. 7:144 10.3389/fnhum.2013.00144 23630479 PMC3633947

[B10] McCarthy-Jones S. Krueger J. Larøi F. Broome M. Fernyhough C. (2013). Stop, look, listen: the need for philosophical phenomenological perspectives on auditory verbal hallucinations. Front. Hum. Neurosci. 7:127 10.3389/fnhum.2013.00127 23576974 PMC3620561

[B11] Stephane M. (2013). Auditory verbal hallucinations result from combinatoric associations of multiple neural events. Front. Hum. Neurosci. 7:239 10.3389/fnhum.2013.00239 23755004 PMC3668292

[B12] Tucker R. Farhall J. Thomas N. Groot C. Rossell S. L. (2013). An examination of auditory processing and affective prosody in relatives of patients with auditory hallucinations. Front. Hum. Neurosci. 7:531 10.3389/fnhum.2013.00531 24046737 PMC3764330

